# Neuroblastoma Patients’ Outcome and Chromosomal Instability

**DOI:** 10.3390/ijms242115514

**Published:** 2023-10-24

**Authors:** Marzia Ognibene, Patrizia De Marco, Loredana Amoroso, Martina Fragola, Federico Zara, Stefano Parodi, Annalisa Pezzolo

**Affiliations:** 1 U.O.C. Genetica Medica, IRCCS Istituto Giannina Gaslini, 16147 Genova, Italy; patriziademarco@gaslini.org (P.D.M.); federicozara@gaslini.org (F.Z.); 2 U.O.C. Oncologia Pediatrica, IRCCS Istituto Giannina Gaslini, 16147 Genova, Italy; loredanaamoroso@gaslini.org; 3 Epidemiologia e Biostatistica, Direzione Scientifica, IRCCS Istituto Giannina Gaslini, 16147 Genova, Italy; martinafragola@gaslini.org (M.F.); stefanoparodi@gaslini.org (S.P.); 4 IRCCS Istituto Giannina Gaslini, 16147 Genova, Italy; annalisapezzolo56@gmail.com

**Keywords:** neuroblastoma, chromosome instability, aneuploidy, breakpoint instability index

## Abstract

Chromosomal instability (CIN) induces a high rate of losses or gains of whole chromosomes or parts of chromosomes. It is a hallmark of most human cancers and one of the causes of aneuploidy and intra-tumor heterogeneity. The present study aimed to evaluate the potential prognostic role of CIN in NB patients at diagnosis. We performed array comparative genomic hybridization analyses on 451 primary NB patients at the onset of the disease. To assess global chromosomal instability with high precision, we focused on the total number of DNA breakpoints of gains or losses of chromosome arms. For each tumor, an array-CGH-based breakpoint instability index (BPI) was assigned which defined the total number of chromosomal breakpoints per genome. This approach allowed us to quantify CIN related to whole genome disruption in all NB cases analyzed. We found differences in chromosomal breakages among the NB clinical risk groups. High BPI values are negatively associated with survival of NB patients. This association remains significant when correcting for stage, age, and *MYCN* status in the Cox model. Stratified analysis confirms the prognostic effect of BPI index in low-risk NB patients with non-amplified *MYCN* and with segmental chromosome aberrations.

## 1. Introduction

Neuroblastoma (NB) is a pediatric tumor of embryonic origin causing 12% of all pediatric cancer mortality [1,2]. The 5-year overall survival rate for high-risk NB patients is less than 40% [2,3]. NB is a heterogeneous disease, since in some cases, lesions regress spontaneously, while in others, the tumor is very aggressive, manifesting itself in a recurrent refractory and metastatic way [1,2,3,4]. Amplification of the proto-oncogene *MYCN*, present in 25% of NB, is the most important genomic marker for the prognosis and treatment decision [3,5,6]. Advances in pan-genomic analysis of NB samples revealed recurrent segmental chromosomal aberrations [7,8,9,10,11,12,13,14]. Many studies have confirmed that segmental chromosome aberrations (SCAs), the loss or gain of a portion of a chromosome arm, presage unfavorable outcomes for NB patients [7,8,9,10,11,12,13,14]. The overall genomic profile is an important prognostic marker which is taken into account for treatment stratification of NB patients [7]. Two tumor genomic profiles have been identified, which classify NB patients into two subgroups associated with different prognoses: (i) NB with a genomic profile that presents only alterations in the number of whole chromosomes (NCA), which has an excellent prognosis even in older patients with metastatic disease, and (ii) NB with a genomic profile with recurrent segmental chromosomal abnormalities (SCAs) due to a myriad of unbalanced translocations, associated with poor prognosis and multi-drug resistance. The recurrent segmental chromosomal anomalies are the 1p, 3p, 4p, 6q, and 11q losses, including potential tumor suppressor genes not yet identified, as well as the 1q, 2p, and 17q gains, including hypothetical oncogenes. The presence of at least one of these recurrent anomalies predicts the course of the disease. A tumorigenesis model for NB has been proposed in which tumors with a favorable biological behavior and therefore a tendency towards spontaneous regression or maturation exhibit only numerical abnormalities of entire chromosomes [15].

Different classification systems are used to define risk and assign treatment to NB patients [16]. The oldest system is the International Neuroblastoma Staging System (INSS), which was developed in 1986 [17]. The INSS staging system utilizes tumor location, lymph node status, bone marrow assessment, and imaging studies to ascertain metastases to determine whether a NB tumor is stage 1, 2A, 2B, 3, 4, or 4S [17,18]. The International Neuroblastoma Risk Group Staging System (INRGSS) was developed in 2009 and used to stratify NB patients [19,20]. The INRGSS system uses image-defined risk factors and admits four stages: localized disease without image-defined factors (L1) and localized disease with image-defined factors (L2), metastatic disease (M), and metastatic disease in very young children that is restricted to skin, liver, or limited marrow involvement (MS). A combination of some known prognostic factors, such as age at diagnosis, stage, histology, ploidy, *MYCN* amplification, and chromosome 11q loss, is used to assign risk [21]. The revised 2021 COG risk classification system incorporates INRGSS with clinical and biologic prognostic factors, including loss of 1p, 3p, 4p, and 11q and gain of 1q, 2p, and 17q SCA, to classify risk and stratify treatment for NB patients [22]. The revised 2021 COG system allowed for the assignment of NB patients into five prognostic and treatment risk groups: very low, low, intermediate, high-risk, and ultra-high-risk. NB patients with high-risk and ultra-high-risk disease initially respond to treatment, but often relapse and become resistant to therapies [23].

We believe that additional molecular biomarkers such as chromosome instability (CIN) should be studied to determine whether current paradigms could be optimized to achieve greater accuracy in risk assignment. CIN, which is defined as a high rate of losses or gains of whole or partial chromosomes, is a hallmark of most human cancers and the most important cause of tumor aneuploidy and intra-tumor heterogeneity [24,25,26,27,28,29]. CIN leads to an unequal distribution of chromosomes in daughter cells, inducing tumor development and progression [30,31]. CIN is associated with poor patients’ outcome and drug resistance; the latter is probably mediated by evolutionary adaptation promoted by intra-tumor heterogeneity [32,33,34]. CIN is a dynamic state in which cells continuously gain or lose parts or whole chromosomes, and that is why it is the main mediator of aneuploidy [35,36]. The relationship between CIN and prognosis suggests that stratifying tumors according to CIN might help to better define clinical risk assessment [37,38]. The high frequency of both segmental and numerical copy number aberrations in NB cells suggests that CIN is an important feature of NB [7,8,9,10,11,12,13,14,39]. Indeed, since segmental chromosome aberrations are related to tumor growth and aggressiveness, it can be remarked that CIN has a role in NB development and in poor outcomes for the disease.

The aim of the present study was to identify and evaluate the level of chromosomal instability as an additional biomarker that can optimize the revised 2021COG system. The number of both segmental and numerical copy number aberrations can be considered a surrogate marker for CIN, providing only partial information and leading to an underestimation of chromosome instability. Since chromosomal instability depends on breakage events, we measured CIN as the proportion of the altered genome rather than the number or pattern of the alterations. We introduced a quantitative CIN index: a breakpoint instability index (BPI), which defines the total number of chromosomal breakpoints per genome.

## 2. Results

### 2.1. Chromosomal Instability (CIN) in Neuroblastoma Samples

**Figure 1:**
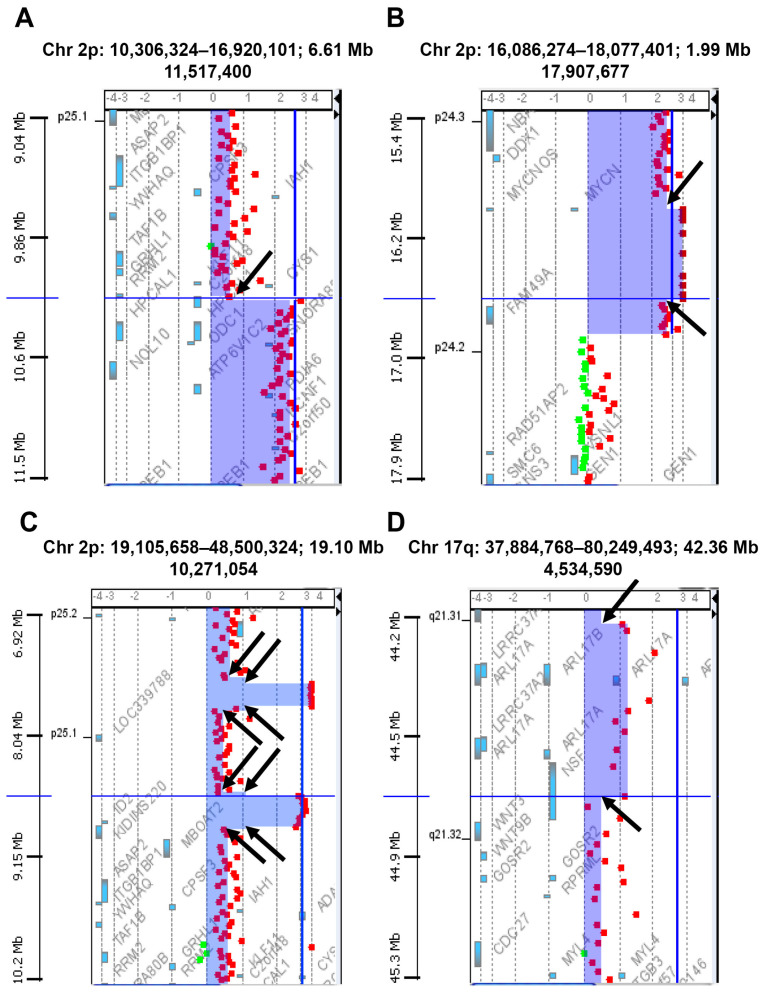
Examples of putative breakpoints in areas of frequent chromosomal rearrangements and of points of steep copy number changes called within larger aberrations in neuroblastoma. Breakpoints on chromosomes 2p (**A**–**C**) and 17q (**D**) are indicated by black arrows. In particular, (**B**) shows two breakpoints within the amplicon containing the *MYCN* oncogene. Each panel shows data points for the log2 ratio of fluorescence between tumor DNA labeled with a red fluorophore (Cyanine 5) and the normal reference DNA labeled with a green fluorophore (Cyanine 3). Shaded areas detect aberrations called by the ADM-1 algorithm. The genes are indicated by blue boxes.

**Figure 2:**
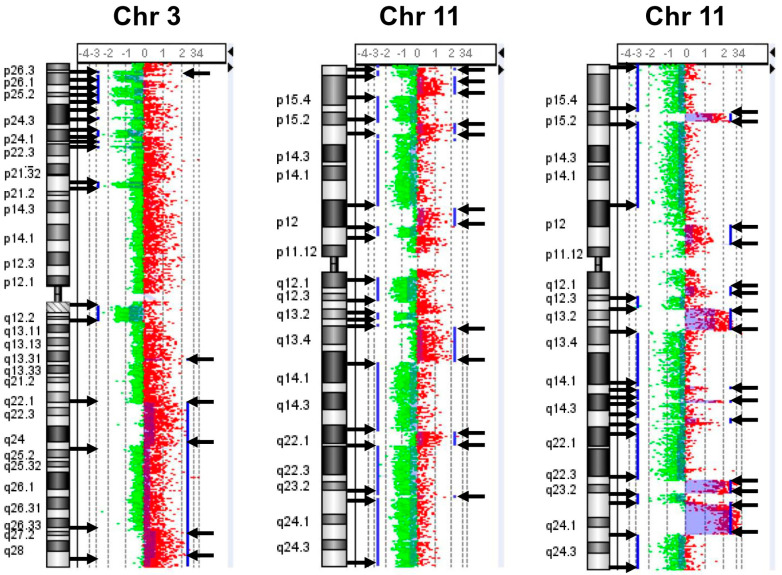
Examples of chromosomes 3 and 11 displaying the characteristic features of chromothripsis in neuroblastoma. Array-CGH ideograms showing chromosome losses (green dots) and amplifications (red dots) identified with the ADM-1 algorithm. Breakpoints are indicated by black arrows. Vertical blue lines indicate DNA losses and gains.

We performed array comparative hybridization (a-CGH) analyses on 451 primary NB samples at the onset of the disease, before any kind of therapeutic treatment. To assess global chromosomal instability with high precision, we focused on the total number of DNA breakpoints of gains or losses of chromosome arms. The beginning and the end of copy number alterations were considered as breakpoints. Furthermore, strong copy number changes found within large aberrations were also defined as breakpoints (Figure 1). For each tumor, a breakpoint instability index (BPI) was assigned which defined the total number of putative breakpoints per genome (Supplementary Table S1). This approach allowed us to quantify chromosomal instability related to whole genome disruption in all the analyzed NB tumors. Looking at all tumors together, the highest number of breaks occurred at chromosomes 2p and 17q, suggesting a particular proclivity of these chromosomes for breaks in NB. We also observed chromothripsis affecting various chromosomes in NB samples. The chromothripsis could be identified with array-CGH by finding regions with multiple altered copy numbers involving tens to hundreds of breakpoints located on a chromosome (the criterion for identifying chromothripsis events is to find at least 10 breakpoints on the same chromosome) [40]. The frequency of chromothripsis was very low (1.1%) and particularly involved chromosomes 2, 3, 11, 12, and 16, with chromosome 11 as the most affected one (Figure 2). We found differences in chromosomal breakages among the various NB clinical risk groups.

### 2.2. Association between BPI Index and Main Prognostic Factors

We observed a clear association between the BPI index and the main negative prognostic factors (stage, age, segmental chromosomal aberrations, and *MYCN* status). Table 1 shows the association between BPI and the main prognostic factors at diagnosis. BPI increased significantly with older age, and it was higher in the M stage and in patients with amplified *MYCN* status, *p* < 0.001. In more detail, a highly statistically significant difference was found when comparing the first age class with both the second (adjusted *p* < 0.001) and the third one (adjusted *p* < 0.001), while the difference between 18–59 and ≥ 60-month patients was marginally statistically significant (adjusted *p* = 0.044).

### 2.3. BPI Values and Survival of NB Patients

**Figure 3:**
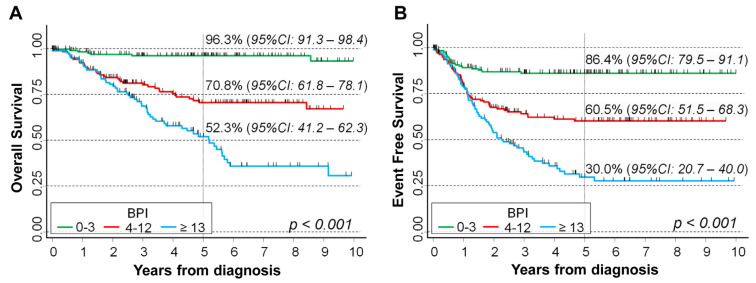
Association between BPI and survival in a cohort of 451 NB patients. Overall survival (**A**) and event free survival (**B**). Five-year survival probabilities are shown. CI: confidence intervals. *p*: *p*-value obtained via the log-rank test for trend.

Considering the association between BPI and patients’ survival, higher values were associated with a poorer prognosis both for overall survival (OS) (Figure 3A) and for event free survival (EFS) (Figure 3B), with clear evidence of a trend (*p* < 0.001 for both OS and EFS, log-rank test for trend). Five-year OS was 52.3% for BPI values ≥ 13, 70.8% for BPI between 4 and 12, and 96.3% for BPI < 4. With regard to EFS, the corresponding figures were 30%, 60.5%, and 86.4%. The median follow up time was 4.0 years for OS and 2.5 years for EFS.

Multivariable analysis via Cox regression model confirmed the observed associations after adjusting for the effect of the considered confounding factors, both for OS (HR = 2.6 and HR = 5.0, comparing the last and the second BPI tertile with the first one, *p* < 0.001) and for EFS (HR = 2.3 and 3.8, respectively, *p* < 0.001, test for trend) (Table 2). The presence of a linear trend was confirmed using the original non-categorized BPI variable as a predictor in the multiple regression model (*p* = 0.001 for OS and *p* = 0.004 for EFS). In the multiple regression analysis, patients in risk group MS were excluded due to insufficient number. The analysis was repeated after excluding patients diagnosed in the first treatment era (i.e., before 1999, *n* = 10). Results are shown in Supplementary Table S2 and are consistent with those reported in Table 2, confirming the observed association between high BPI levels and poor patients’ survival.

**Figure 4:**
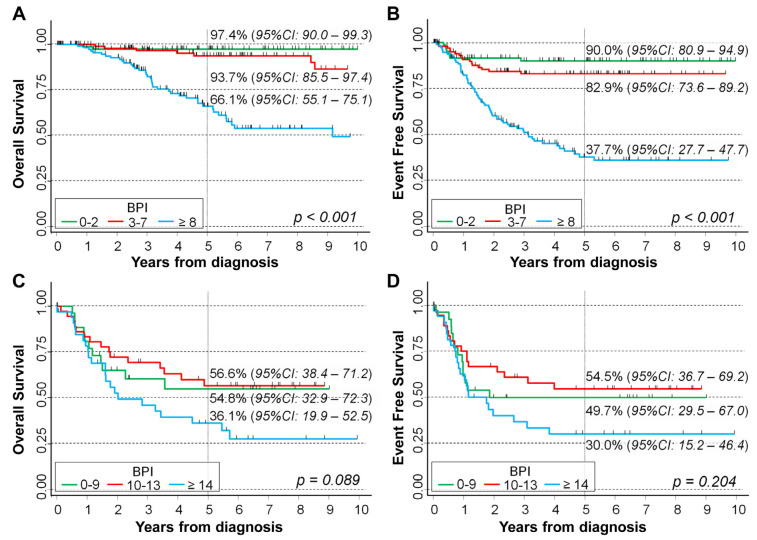
Association between BPI and survival in a cohort of 451 NB patients stratified by *MYCN* status. Normal *MYCN* (**A**,**B**) and amplified *MYCN* (**C**,**D**). Five-year survival probabilities are shown. CI: confidence intervals. *p*: *p*-value obtained via the log-rank test for trend.

Results of the survival analysis stratified by *MYCN* status are shown in Figure 4. A clear inverse association between BPI levels and patient survival was observed in patients with non-amplified *MYCN*, both for OS (Figure 4A, *p* < 0.001) and for EFS (Figure 4B, *p* < 0.001, test for trend). Five-year OS was 66.1% in patients with BPI ≥ 8, 93.7% for BPI between 3 and 7, and 97.4% for BPI between 0 and 2. The corresponding figures for EFS were 37.7%, 82.9%, and 90%, respectively. In patients with *MYCN* amplification, no statistically significant association was found (*p* = 0.089 for OS and *p* = 0.204 for EFS) (Figure 4C,D). The median follow up time for the OS was 4.6 years in patients with non-amplified *MYCN* and 2.5 years for patients with amplified *MYCN*. The corresponding values for EFS were 3.0 and 1.8 years, respectively.

The observed association in patients with non-amplified *MYCN* status was confirmed with multivariable regression analysis both for OS and for EFS (Table 3). With regard to OS, adjusted HRs were 2.0 in the second tertile of BPI and 4.9 in the last tertile, compared with the first one (*p* = 0.002). The corresponding estimates for EFS were 1.6 and 5.5, respectively. This trend was also observed when the original non-categorized BPI values were included in the regression model (*p* = 0.002 for OS and *p* = 0.001 for EFS). Analysis restricted to patients diagnosed in the second and third treatment era showed a consistent pattern (Supplementary Table S3).

Analysis with the Cox regression model confirmed the lack of association between BPI levels and both OS (*p* = 0.122) and EFS (*p* = 0.264) in patients with amplified *MYCN*, even after adjusting for the potential confounding of the considered prognostic factors (Table 4) and after excluding patients diagnosed during the first treatment era (Supplementary Table S4).

**Figure 5:**
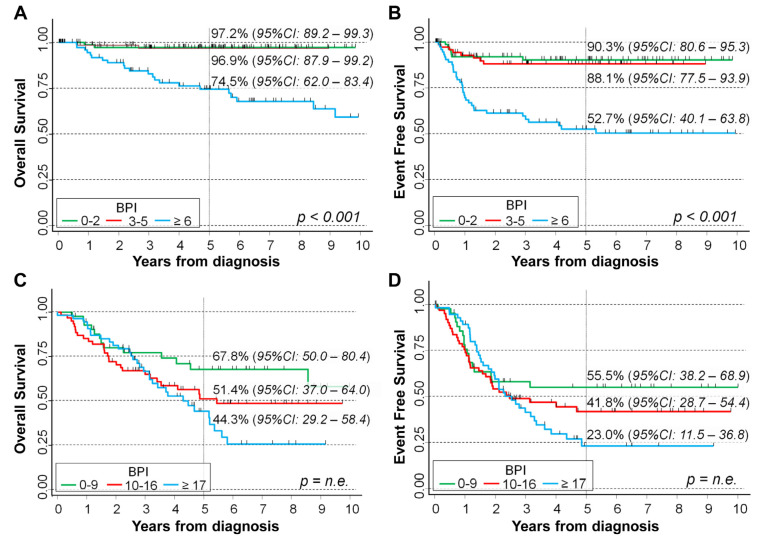
Association between BPI and survival in a cohort of 451 NB patients by stage at diagnosis: localized stage (**A**,**B**) and stage M (**C**,**D**). Five-year survival probabilities are shown. CI: confidence intervals. *p*: *p*-value obtained via the log-rank test for trend. n.e.: not evaluable due to the violation of the proportional hazard’s assumption.

In Figure 5, we analyze patients’ survival stratified by stage at diagnosis. In localized stages, higher BPI levels were associated with a poor survival both for OS (*p* < 0.001, Figure 5A) and for EFS (*p* < 0.001, Figure 5B). Five-year OS was 74.5% in patients with BPI ≥ 6, 96.9% for BPI between 3 and 5, and 97.2% for BPI < 3 (*p* < 0.001). As for EFS, the corresponding figures were 52.7%, 88.1%, and 90.3%, respectively (*p* < 0.001). In stage M patients, BPI values above the second tertile seemed associated with a poorer outcome, but the crossing of the corresponding curves indicated a violation of the proportional hazard assumption both for OS (Figure 5C) and for EFS (Figure 5D), which prevented statistical inference with the log-rank test. The median follow up time was 4.2 for localized stages, 3.2 years for stage M, and 5.3 years for stage MS patients. The corresponding values for EFS were 3.8, 2.1, and 2.7 years, respectively.

Analyses via Cox regression model confirmed the negative association between high BPI levels and survival in patients with localized NB for both OS and EFS, which remains statistically significant after adjusting for the potential confounding effect of the main available prognostic factors (Table 5). Adjusted HRs in the second and third tertile of the BPI distribution were 1.1 and 6.3 for OS (*p* = 0.002) and 1.2 and 5.5 for EFS (*p* < 0.001). The trend was confirmed when the original non-categorized BPI values were included in the regression model (*p* < 0.001 for both OS and EFS) after excluding patients diagnosed in the first treatment era (Supplementary Table S5).

In stage M patients, a clear association was not found, but the violation of the proportional hazard assumption prevented us from performing statistical inference. When BPI original values were introduced in the Cox regression models, no statistically significant association emerged for either OS (*p* = 0.090) or EFS (*p* = 0.464) (Table 6) after excluding patients diagnosed in the first treatment era (Supplementary Table S6).

### 2.4. Assessing the Independent Potential Prognostic Role of BPI Index in Patients with Cromosome Abnormalities

**Figure 6:**
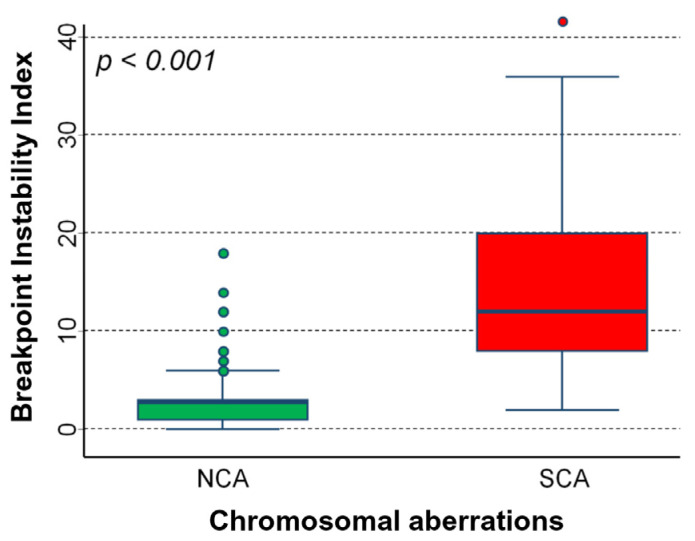
Chromosome-breakage genomic instability in NB with a genomic profile with recurrent segmental chromosomal aberrations (SCAs) and NB tumors with a genomic profile that presents only alterations in the number of whole chromosomes (NCA). Box plot of data representing genomic instability calculated as a total number of chromosomal putative breakpoints per genome (BPI). Green box: numerical chromosomal aberrations. Red box: segmental chromosomal aberrations. *p*: *p*-value obtained with the Mann–Whitney U test.

Figure 6 shows the association between BPI and the type of chromosomal aberrations in the analyzed cohort of 451 NB patients. BPI was clearly higher in patients carrying SCA rather than in those with NCA (*p* < 0.001).

**Figure 7:**
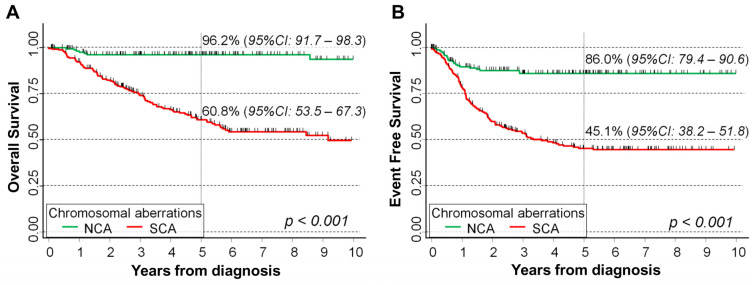
Association between type of chromosomal aberrations in the primary tumors at diagnosis and survival in a cohort of 451 NB patients. Overall survival (**A**) and event free survival (**B**). Five-year survival estimates probabilities are shown. NCA: numerical chromosomal aberrations. SCA: segmental chromosomal aberrations. *p*: *p*-value obtained with the log-rank test.

As expected, survival of patients with SCA was clearly poorer than that of patients carrying NCA in their primary tumor (*p* < 0.001 for both OS (Figure 7A) and EFS (Figure 7B)). OS was 96.2% in patients with NCA and 60.8% in patients with SCA (Figure 7A), while the corresponding estimates for EFS were 86.0% and 45.1%, respectively (Figure 7B).

**Figure 8:**
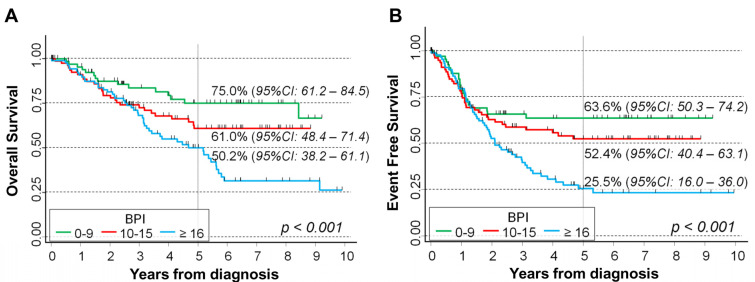
Association between BPI and survival in a cohort of 261 NB patients with segmental chromosomal aberrations in the primary tumors at diagnosis: overall survival (**A**) and event free survival (**B**). Five-year survival probabilities are shown. *p*: *p*-value obtained by the log-rank test for trend.

Figure 8 shows the OS and EFS analysis of the 261 NB patients with SCA, stratified via BPI levels. A clear and statistically significant trend was observed for OS, with higher values of BPI corresponding to a poorer survival (50.2% in patients with BPI ≥ 16, 61% in patients with BPI between 10 and 15, and 75% in patients with BPI below 10, respectively, *p* < 0.001, Figure 8A). A similar pattern was observed when analyzing the EFS (25.5% in the group with highest BPI, 52.4% in correspondence with intermediate values, and 63.6% for the highest values, *p* < 0.001, Figure 8B).

The Cox regression model, adjusted for the potential confounding of *MYCN* status, age, stage, and treatment era, confirmed the observed trend for both OS and EFS (Table 7), even if the association with the original non-categorized BPI values was statistically borderline for OS (*p* = 0.049) and no longer significant for EFS (*p* = 0.074). In more detail, the adjusted HR estimates for OS were 1.2 for BPI between 10 and 15 and 2.2 for BPI ≥ 16, compared to BPI < 10 (*p* = 0.006). A similar pattern was observed for EFS, where the corresponding values were 1.2 and 1.9, respectively (*p* = 0.008). Consistent results were observed when restricting the analyses to patients diagnosed in the second and third treatment era (Supplementary Table S7).

Unfortunately, no survival analysis via BPI levels was feasible among patients carrying NCA due to the small number of observed outcomes (seven deaths and 22 events).

## 3. Discussion

Many studies have led to the general conclusion that CIN is a marker of aggressive tumors [41,42,43,44,45]. CIN, defined by the presence of multiple chromosomal abnormalities, contributes to intra-tumor heterogeneity, tumor progression, drug resistance, and treatment failure [31,32,33,34]. Chromosome breakage followed by defective DNA repair leads to the formation of chromosomal abnormalities, resulting in amplifications and deletions of oncogene or tumor suppressor genes in tumors [36,37]. NB, like many other tumors, is considered a genetic disease, as it presents peculiar mutations, a telomere maintenance mechanism, and numerical and segmental chromosomal aberrations [7,8,9,10,11,12,13,14]. Some studies have demonstrated evidence of CIN in NB cells. It has been reported that some genes involved in the processes of chromosome replication, regulation of chromatin state, and chromosome segregation are over-expressed in NB cells [46]. Notably, many NB predisposing variants occur in genes involved in the control of genome stability, mitosis, and chromosome separation [39]. A study identified the chromosomal instability gene *USP24* as frequently deleted in NB [47]. The authors suggested that *USP24* is a tumor suppressor gene which maintains the stability of the genome, preventing aneuploidy by maintaining spindle-associated CRMP2, which is required for mitotic precision.

NB has two distinct patterns of CIN: (i) numerical chromosome aberrations (whole chromosome aneuploidy) caused by microtubule destabilization that leads to chromosome segregation errors, which is associated with good prognosis; and (ii) recurrent segmental chromosome aberrations, caused by unbalanced chromosomal translocations, which in turn are related to a defect in the DNA repair, which is a powerful negative prognostic factor [48]. However, the mechanisms behind the chromosomal instabilities in NB have not yet been elucidated. To address open questions about NB and CIN, we performed array comparative genomic hybridization (a-CGH) analysis in 451 primary tumors from patients displaying NB at the onset of the disease. Here, we described a novel approach for a global analysis of chromosomal rearrangements in NB, the breakpoint analysis, which led us to define an instability index (BPI) starting from array-CGH data, which include all chromosomal breaks within the genome. Breakpoints deduced from array-CGH data have previously been used to assess genomic instability in breast cancer [49,50]. To quantify CIN with high precision, we focused on breakpoints located at the beginning and at the end of copy number gains and losses, including chromosomal breaks within larger aberrations. We identified the number of chromosomal breakpoints and the pattern of breaks on individual chromosomes in each NB tumor observed with array-CGH. A BPI was assigned to each tumor which included all significant chromosomal breaks in individual chromosomes. We considered the BPI value as a measure of chromosomal instability. Looking at all tumors together, the highest density of breaks occurred at chromosomes 2p and 17q, which have been shown to lead to recurrent aberrations, suggesting a particular tendency of these chromosomes towards instability in NB. Genome sequencing experiments of solid tumors including NB have revealed the existence of complex genomic rearrangements involving tens or hundreds of breakpoints that appear to have arisen through a single catastrophic event called chromothripsis [51,52]. The fragmentation of the chromatids and subsequent reassembly give rise to chromothripsis, which apparently represents an important mechanism of carcinogenesis, distinct from the progressive accumulation of chromosomal aberrations. Although the underlying cause of chromothripsis is not fully understood, several hypotheses have been proposed (ionizing radiation, telomere attrition, abortive apoptosis, and premature chromosome condensation) [52,53,54]. We observed that chromothripsis in NB particularly affected chromosomes 2, 3, 11, 12, and 16 in high-risk disease. This suggests that chromosomal instability is not the same for all chromosomes and that the contribution of each chromosome to NB development could be determined by their structural characteristics.

Notably, BPI increased significantly in patients with amplified *MYCN* status. BPI was clearly higher in NB carrying segmental chromosome aberrations rather than in those with only numerical chromosome aberrations. A model has been proposed by which aggressive NB with segmental chromosome alterations may arise from an intermediate stage characterized by whole chromosome aneuploidy [55]. Thus, aneuploidy and the consequent presence of alteration in the number of whole chromosomes may be a first step leading to the development of segmental chromosome aberrations. As proof of this theory, we have recently described a NB case that progressed from stage 4S (corresponding to stage MS in the INRGSS classification) to stage 4 (stage M in the INRGSS classification) with a very poor outcome. This patient had only numerical copy number aberrations in the primary tumor at stage 4S and acquired further segmental chromosomal alterations during progression to true stage 4 [56]. It has been shown that numerical chromosome aberrations can lead to severe DNA damage or chromothripsis, resulting in segmental chromosome alterations [57]. In unfavorable NB, whole chromosome aneuploidy leads to the subsequent development of segmental chromosomal alterations, favoring a multistep model of NB progression.

Using the BPI analysis approach, we established the variability in degrees of chromosomal instability within each NB risk group. We found differences in the total number of chromosomal breakpoints per genome between the localized (L1 and L2 combined) and the metastatic NB risk groups. High-risk NB showed the highest density of breaks compared to other risk groups, suggesting that chromosomal instability is associated with more aggressive NB. High BPI values are negatively associated with both overall survival and event free survival of NB patients. This association remains significant when correcting for stage, age, and *MYCN* status in the Cox model. Stratified analysis confirms the prognostic effect of BPI index in NB patients with low-risk disease and with non-amplified *MYCN*.

Further studies on large independent cohorts will be necessary to further validate our results; however, this study strongly points out BPI as a potential poor prognostic biomarker in NB. CIN appears higher in the more aggressive NB. The proportion of numerical and segmental aberrations differs between localized and metastatic NB, with a higher preponderance of segmental aberrations in metastatic tumors. This observation suggests that CIN might be a major player in the oncogenesis of NB.

## 4. Materials and Methods

### 4.1. Neuroblastoma Samples

Primary NB samples were stored in the BIT-Gaslini Biobank, Tissue Section, IRCCS Istituto G. Gaslini, Genova, Italy. DNAs were extracted using a QIAamp DNA Extraction Kit (Qiagen, Hilden, Germany), according to the manufacturer’s instructions. Between 1995 and 2020, 451 samples of primary tumors from NB patients were collected and included in the analyses. Follow up data were obtained from the Italian Neuroblastoma Registry database, which includes all patients with peripheral neuroblastic tumors diagnosed at the institutions participating in the Italian Neuroblastoma Group [58]. The structure and protocol of the Italian Neuroblastoma Registry were approved by the Ethics Committees of each participating center. The enrolled patients or their parents/guardians were asked to sign an informed consent form. The Italian Neuroblastoma Registry database is stored in a secure server of the Italian Inter-University Consortium CINECA (Casalecchio di Reno, Italy) that can be accessed only by authorized users. It received the ISO 9001:2015 Quality Management System certification and the ISO/IEC 27,001:2013 Information Security Management System certification.

### 4.2. Genomic Profile Analysis

Copy number alterations in tumor DNA were determined relative to sex-matched normal human DNA (Agilent Technologies, Santa Clara, CA, USA) and were identified with array-CGH analysis using microarray slides (180 K) which contained 180.000 oligonucleotide probes (Agilent Technologies). For sample preparation and hybridization, we followed the manufacturer’s protocol. Briefly, amplified DNA was labeled with random priming using either Cy5-dUTP (tumor DNA) or Cy3-dUTP (normal reference DNA). Following purification with Microcon Centrifugation Filters, Ultracel YM-30 (Millipore, Burlington, MA, USA), probes were denatured and pre-annealed with 50 μg of human Cot-1 DNA (Thermo Fisher Scientific, Waltham, MA, USA). Hybridization was performed at 67 °C for 24 h with constant rotation.

After hybridization, slides were washed according to the manufacturer’s instructions and scanned immediately with a DNA Microarray Scanner (Agilent Technologies). Each hybridization produced a pair of 16-bit images, which were processed using Agilent Feature Extraction 10.5 Software. The data were analyzed using the Genomic Workbench 7.0.40 software (Agilent); the altered chromosomal regions and breakpoint events were detected using ADM-1 (threshold 10) with a 0.5 Mb window size to reduce false positives [10]. The cutoffs to call aberrations were defined at loci with log2 ratio ≥ 0.2 for the gains, log2 ratio ≤ −0.3 for losses, and log2 ratio ≤ −2 for homozygous deletions. Amplifications were defined at loci with log2 ratio ≥ 2, and loci with log2 ratio ≥ 3.5 were considered to represent a high level of amplifications (more than 50 copies of the gene). Chromosome positions were determined using GRCh38/hg19 (UCSC Genome Browser, http://genome.ucsc.edu, February 2009 release, NCBI Build 37.1, accessed on 20 June 2022). Only chromosomal copy number variations with a frequency < 5% present in the Database of Genomic Variants (DGV: http://projects.tcag.ca/variation/, accessed on 20 June 2022) were taken into consideration. The raw data were stored in the BIT-Gaslini Biobank, Genomic Section, IRCCS Istituto G. Gaslini, Genova.

Breakpoints were identified as genomic locations where a change in copy number had occurred, as determined by ADM-1. A genomic position was considered a breakpoint if the Log2 value alteration between two adjacent segments from centromere to telomere was >0.3 [59,60].

Written informed consent was obtained from the parents to report the case of their child, in accordance with the Declaration of Helsinki. This study was approved by the Italian Institutional Ethics Committee (measure no. 270/17 related to the clinical study protocol IGG-NCA-AP-2016, approved on 15 December 2016 and renewed on 24 May 2021).

### 4.3. Patients’ Characteristics

The main clinical and demographic characteristics of the 451 patients included in the study are listed in Table 8. Male gender was slightly prevalent (55%), most patients were diagnosed before 18 months of age (51% vs. 38% in age 18–59 months vs. 11% in the age class ≥ 60 months, respectively). The majority of patients were diagnosed in localized stages (38% in stage L1 and 19% in stage L2, respectively), with 38% in stage M. Only 20 patients were observed in stage MS (5%), and they were excluded from stratified analyses due to the insufficient sample size. Most patients had a non-amplified *MYCN* status (77%). Thirty-two percent experienced at least one event, and 22% eventually died. The 143 observed events included the following: 133 disease relapses or progressions (34 in local sites, 30 combined, 63 metastatic, and 6 not specified), 90 of whom eventually died for NB, and 1 for a secondary tumor (a thyroid papillary carcinoma); 1 death for a secondary tumor (a precursor B-cell lymphoblastic leukemia) in a not-relapsed patient; 2 secondary tumors in patients who neither relapsed nor died (both acute myeloid leukemia); 1 death for macrophage activation syndrome; 5 deaths for treatment toxicity; and 1 death for unknown cause.

### 4.4. Statistical Analysis

Descriptive statistics were reported as absolute frequencies and percentages for categorical variables and as median values and interquartile ranges (IQRs) for quantitative variables. The association between BPI and the available prognostic factors was assessed with the Mann–Whitney U test and Kruskal–Wallis’s test in the presence of more than two categories. Sub-group comparisons were performed, adjusting the corresponding *p*-value via the Bonferroni’s correction.

The potential prognostic role of BPI was evaluated both for overall (OS) and event free survival (EFS). OS time was calculated as the difference between the date of the last follow up or death and the date of diagnosis. EFS time was obtained from the date of diagnosis to the last follow up or event, which included the first occurrence of relapse, progression, secondary tumor, or death. Accordingly, in the OS analysis, each patient contributed either a censored time if he/she was alive at the last available follow up, independently of his/her disease status (i.e., not considering the response to the therapies or the occurrence of a relapse), or a complete time in case of death. With regard to EFS, complete follow up times were calculated using the same approach, but for living patients who experienced an event (i.e., a disease recurrence or a secondary tumor), the corresponding time was shortened to the first occurrence of the event and considered as complete. Follow up data assessing the living status (including the cause of death for deceased patients) and the occurrence of disease progression/relapse or occurrence of a secondary malignancy were required yearly from each center. Follow up times for patients who did not experience any event were censored at the last available date or at the end of the last follow up update of the Italian Neuroblastoma Registry (15 February 2023).

Both OS and EFS were estimated via the Kaplan–Meier method, and differences between groups were assessed with the log-rank test and the log-rank test for trend when appropriate [61]. Patients in the whole cohort and in each subgroup were split into three groups based on the tertile values of BPI distribution in order to obtain a quite homogeneous sample size in each analysis. As a consequence, the identified BPI cut-offs could vary in the different sub-cohorts under study. Kaplan–Meier five-year survival estimates of OS and EFS and their related 95% confidence intervals (95%CI), obtained via the Kalbfleisch and Prentice method [62], were also provided to allow comparisons with both internal and external data (i.e., published results from other cohorts). The original non-categorized BPI variable was also analyzed to assess a linear association between BPI values and both OS and EFS. The association between BPI and patients’ survival, adjusted for the effect of potential confounders (age, treatment era, *MYCN* status, and stage at diagnosis), was evaluated with the multivariable Cox regression model. Three treatment eras were identified, similarly to other investigations on the same cohort [58], by the following periods of diagnosis: <1999 (*n* = 10), 1999–2009 (*n* = 58), and ≥2010 (*n* = 383). The first cut-off (1999) corresponded to the centrally revised pathology, while the second cut-off (2009) corresponded to the introduction of the INRGSS system staging and the introduction of the SIOPEN high-risk treatment protocol. Statistical significance was obtained via the likelihood ratio test and the likelihood ratio test for trend in the presence of more than two groups. Because both *MYCN* status and advanced stage at diagnosis can identify different groups of patients with different genetic characteristics, including CIN, subgroup analyses were also performed, including stratification by *MYCN* status (amplified vs. not amplified tumors) and stage at diagnosis (localized vs. metastatic). Finally, the association between patients’ survival and BPI was also evaluated, restricting the analyses to patients with primary tumors carrying SCA in order to evaluate the potential contribution to BPI as a prognostic factor independent from other CIN. Restriction to patients with NCA was not feasible due to the small number of observed outcomes.

All statistical analyses were performed with the STATA for Windows statistical package (release 13.1, Stata Corporation, College Station, TX, USA).

## 5. Conclusions

In summary, we found that chromosome instability was different in each clinical NB risk group using an array-CGH-based instability index representative of the total number of breakpoints throughout the tumor genome. Chromosomes 2p and 17q were the most frequently broken chromosomes in all clinical risk groups, revealing a particular propensity of these chromosomes to instability in NB. These results indicate that chromosomal breakage events occur gradually, targeting a few specific chromosomes. In this regard, the phenomenon of chromothripsis with a very low frequency that we observed might seem in contrast with our thesis, but anyway, it reflects many breakage events, typical of a chromosomal instability context. The relationship between CIN and prognosis suggests that stratifying NB according to the BPI value might help us to better define clinical risk assessment.

## Figures and Tables

**Table 1:** Association between BPI and the main prognostic factors at diagnosis.

Prognostic Factors	*N*	Median	IQR	*p*
**Age at diagnosis**				<0.001
<18 months	232	3	2–8	
18–59 months	169	12	8–18	
≥60 months	50	18	8–24	
**INRG Stage**				<0.001
L1	166	3	2–6	
L2	81	4	3–12	
M	166	12	8–20	
MS	20	3.5	1–5.5	
***MYCN* status**				<0.001
Non amplified	328	4	2–12	
Amplified	100	12	8–16	

IQR: Interquartile range; *p*: *p*-value obtained with the Kruskal–Wallis test.

**Table 2:** Overall and event free survival of 451 NB patients in relation to the BPI levels evaluated with the Cox model.

		Univariable Analysis			Multivariable Analysis		
BPI	N/O	HR	95%CI	*p*	HR	95%CI	*p*
**Overall Survival**				<0.001 ^#^			<0.001 ^#^
0–3	167/6	1 (ref.)	-		1 (ref.)	-	
4–12	160/39	7.5	3.2–17.8		2.6	0.99–6.7	
≥13	124/55	16.5	7.1–38.4		5.0	2.0–13.0	
Original variable	451/100	1.08	1.06–1.09	<0.001	1.04	1.02–1.07	0.001
**Event Free Survival**				<0.001 ^#^			<0.001 ^#^
0–3	167/19	1 (ref.)	-		1 (ref.)	-	
4–12	160/55	3.4	2.0–5.7		2.3	1.2–4.3	
≥13	124/69	6.4	3.8–10.6		3.8	1.9–7.3	
Original variable	451/143	1.06	1.04–1.08	<0.001	1.03	1.01–1.05	0.003

N/O: Number of patients/outcome (deaths or events). HR: hazard ratio. Multivariable analysis: HRs are adjusted by *MYCN* status, age, stage at diagnosis, and treatment era. Ref.: referent group. Original variable: original values of BPI non-categorized to assess the linear trend. ^#^: *p*-value obtained via likelihood ratio test for trend. In the multivariable analysis, stage MS patients were excluded due to insufficient sample size.

**Table 3:** Overall and event free survival of 328 patients with non-amplified *MYCN* status in relation to the BPI levels evaluated with the Cox model.

		Univariable Analysis			Multivariable Analysis		
BPI	N/O	HR	95%CI	*p*	HR	95%CI	*p*
**Overall Survival**				<0.001 ^#^			0.002 ^#^
0–2	92/2	1 (ref.)	-		1 (ref.)	-	
3–7	111/7	2.7	0.55–12.8		2.0	0.39–10.0	
≥8	125/41	17.3	4.2–71.7		5.3	1.2–24.2	
Original variable	328/50	1.10	1.07–1.12	<0.001	1.05	1.02–1.08	0.002
**Event Free Survival**				<0.001 ^#^			<0.001 ^#^
0–2	92/8	1 (ref.)	-		1 (ref.)	-	
3–7	111/16	1.6	0.70–3.8		1.6	0.67–4.0	
≥8	125/65	7.3	3.5–15.3		5.5	2.3–13.4	
Original variable	328/89	1.07	1.05–1.09	<0.001	1.04	1.02–1.07	0.001

N/O: Number of patients/outcome (deaths or events). HR: hazard ratio. Multivariable analysis: HRs are adjusted by age, stage at diagnosis, and treatment era. Ref.: referent group. Original variable: original values of BPI non categorized to assess the linear trend. ^#^: *p*-value obtained via likelihood ratio test for trend. In the multivariable analysis, stage MS patients were excluded due to insufficient sample size.

**Table 4:** Overall and event free survival of 100 patients with amplified *MYCN* status in relation to the BPI levels evaluated with the Cox model.

		Univariable Analysis			Multivariable Analysis		
BPI	N/O	HR	95%CI	*p*	HR	95%CI	*p*
**Overall Survival**				0.087 ^#^			0.122 ^#^
0–9	27/11	1 (ref.)	-		1 (ref.)	-	
10–13	37/15	0.87	0.40–1.9		0.85	0.38–1.9	
≥14	36/22	1.7	0.84–3.6		1.7	0.81–3.6	
Original variable	100/48	1.03	0.99–1.07	0.168	1.02	0.98–1.06	0.313
**Event Free Survival**				0.202 ^#^			0.264 ^#^
0–9	27/13	1 (ref.)	-		1 (ref.)	-	
10–13	37/16	0.79	0.38–1.7		0.75	0.35–1.6	
≥14	36/22	1.5	0.74–2.9	0.329	1.4	0.70–2.9	
Original variable	100/51	1.02	0.98–1.05	0.329	1.01	0.97–1.05	0.558

N/O: Number of patients/outcome (deaths or events). HR: hazard ratio. Multivariable analysis: HRs are adjusted by age, stage at diagnosis, and treatment era. Ref.: referent group. Original variable: original values of BPI non-categorized to assess the linear trend. ^#^: *p*-value obtained via likelihood ratio test for trend. In the multivariable analysis, stage MS patients were excluded due to insufficient sample size.

**Table 5:** Overall and event free survival of 247 NB patients with localized stage (L1 and L2 combined) at diagnosis in relation to the BPI levels evaluated with the Cox model.

		Univariable Analysis			Multivariable Analysis		
BPI	N/O	HR	95%CI	*p*	HR	95%CI	*p*
**Overall Survival**				<0.001 ^#^			0.002 ^#^
0–2	84/2	1 (ref.)	-		1 (ref.)	-	
3–5	80/2	1.0	0.14–7.1		1.1	0.16–7.9	
≥6	83/24	11.9	2.8–50.4		6.3	1.4–28.6	
Original variable	247/28	1.12	1.08–1.16	<0.001	1.10	1.04–1.15	<0.001
**Event Free Survival**				<0.001 ^#^			<0.001 ^#^
0–2	84/7	1 (ref.)	-		1 (ref.)	-	
3–5	80/8	1.2	0.43–3.3		1.2	0.44–3.4	
≥6	83/35	6.0	2.7–13.6		5.5	2.3–13.3	
Original variable	247/50	1.10	1.07–1.13	<0.001	1.10	1.05–1.14	<0.001

N/O: Number of patients/outcome (deaths or events). HR: hazard ratio. Multivariable analysis: HRs are adjusted by *MYCN* status, age at diagnosis, and treatment era. Ref.: referent group. Original variable: original values of BPI non-categorized to assess the linear trend. ^#^: *p*-value obtained via likelihood ratio test for trend.

**Table 6:** Overall and event free survival of 166 NB patients with stage M at diagnosis in relation to the BPI levels evaluated with the Cox model.

		Univariable Analysis			Multivariable Analysis		
BPI	N/O	HR	95%CI	*p*	HR	95%CI	*p*
**Overall Survival**				n.e.			n.e.
0–9	42/13	1 (ref.)	-		1 (ref.)	-	
10–16	66/28	1.7	0.88–3.3		1.5	0.75–2.8	
≥17	58/31	2.2	1.1–4.2		2.3	1.1–4.5	
Original variable	166/72	1.02	1.00–1.05	0.077	1.02	1.00–1.05	0.090
**Event Free Survival**				n.e.			n.e.
0–9	42/18	1 (ref.)	-		1 (ref.)	-	
10–16	66/34	1.4	0.77–2.4		1.2	0.67–2.1	
≥17	58/38	1.6	0.91–2.8		1.5	0.81–2.7	
Original variable	166/90	1.01	0.99–1.04	0.257	1.00	0.98–1.03	0.464

N/O: Number of patients/outcome (deaths or events). HR: hazard ratio. Multivariable analysis: HRs are adjusted by *MYCN* status, age at diagnosis, and treatment era. Ref.: referent group. Original variable: original values of BPI non-categorized to assess the linear trend. n.e.: not evaluable due to the violation of the proportional hazards’ assumption.

**Table 7:** Overall and event free survival of 261 NB patients with segmental chromosomal aberrations at diagnosis in relation to the BPI levels evaluated with the Cox model.

		Univariable Analysis			Multivariable Analysis		
BPI	N/O	HR	95%CI	*p*	HR	95%CI	*p*
**Overall Survival**				<0.001 ^#^			0.006 ^#^
0–9	71/15	1 (ref.)	-		1 (ref.)	-	
10–15	88/29	1.7	0.91–3.2		1.2	0.61–2.2	
≥16	102/49	2.9	1.6–5.2		2.2	1.2–4.0	
Original variable	261/93	1.04	1.02–1.06	0.001	1.03	1.00–1.06	0.049
**Event Free Survival**				<0.001 ^#^			0.008 ^#^
0–9	71/23	1 (ref.)	-		1 (ref.)	-	
10–15	88/37	1.4	0.81–2.3		1.2	0.68–2.1	
≥16	102/61	2.3	1.4–3.7		1.9	1.1–3.3	
Original variable	261/121	1.03	1.01–1.05	0.003	1.02	1.00–1.05	0.074

N/O: Number of patients/outcome (deaths or events). HR: hazard ratio. Multivariable analysis: HRs are adjusted by *MYCN* status, stage, age at diagnosis, and treatment era. Ref.: referent group. Original variable: original values of BPI, non-categorized to assess the linear trend. ^#^: *p*-value obtained via likelihood ratio test for trend. In the multivariable analysis, stage MS patients were excluded due to insufficient sample size.

**Table 8:** Main characteristics at diagnosis, number of observed deaths, and events of the 451 NB patients included in the analyses.

Patients’ Characteristics	*N*	% ^‡^
**Gender**		
Males	248	55.0
Females	203	45.0
**Age at diagnosis**		
<18 months	232	51.4
18–59 months	169	37.5
≥60 months	50	11.1
**INRG Stage**		
L1	166	38.3
L2	81	18.7
M	166	38.3
MS	20	4.6
Missing	18	4.0
***MYCN* status**		
Non amplified	328	76.6
Amplified	100	23.4
Missing	23	5.1
**Events**	143	31.7
**Deaths**	100	22.2

^‡^: Percentages were calculated after excluding missing data. Events included number of deaths, disease relapse/progressions, and secondary tumors.

## Data Availability

Data are contained within the article or Supplementary Materials.

## Supplementary Materials

The following supporting information can be downloaded at https://www.mdpi.com/article/10.3390/ijms242115514/s1.
