# Supplementary material for: Neuroblastoma Patients’ Outcome and Chromosomal Instability
Source: Int J Mol Sci. 2023 Oct 24;24(21):15514. doi: 10.3390/ijms242115514 (PMC10648898; doi:10.3390/ijms242115514)
Supplement: Supplementary file 1 [file ijms-24-15514-s001.zip › ijms-2626744-supplementary/Supplementary Tables S2-S7.pdf]

Supplementary Tables

Supplementary Table S2. Overall and Event Free Survival of 441 NB patients diagnosed between 1999 and 2020, in relation to the BPI levels evaluated by the Cox model.

		Univariable analysis			Multivariable analysis		
**BPI**	**N/O**	**HR**	**95%CI**	***p***	**HR**	**95%CI**	***p***
**Overall Survival**				< 0.001			< 0.001
0 – 3	161/4	1 (ref.)	-		1 (ref.)	-	
4 – 12	157/38	10.8	3.8 – 30.2		3.5	1.1 – 10.8	
≥ 13	123/55	24.5	8.9 – 67.9		7.3	2.4 – 22.6	
Original variable	441/97	1.08	1.06 – 1.10	< 0.001	1.04	1.02 – 1.07	< 0.001
**Event Free Survival**				< 0.001			< 0.001
0 – 3	161/16	1 (ref.)	-		1 (ref.)	-	
4 – 12	157/54	3.9	2.2 – 6.9		2.7	1.4 – 5.4	
≥ 13	123/68	7.4	4.3 – 12.8		4.3	2.1 – 8.7	
Original variable	441/138	1.06	1.05 – 1.08	< 0.001	1.03	1.01 – 1.05	0.005

N/O: Number of patients/Outcome (Deaths or Events). HR: Hazard Ratio. Multivariable analysis: HRs are adjusted by *MYCN* status, age, and stage at diagnosis. ref. = referent group. Original variable = original values of BPI non categorized to assess the linear trend.

Supplementary Table S3. Overall and Event Free Survival of 318 patients diagnosed between 1999 and 2020 with not amplified *MYCN* status in relation to the BPI levels evaluated by the Cox model.

		Univariable analysis			Multivariable analysis		
**BPI**	**N/O**	**HR**	**95%CI**	***p***	**HR**	**95%CI**	***p***
**Overall Survival**				< 0.001			0.006
0 – 2	89/1	1 (ref.)	-		1 (ref.)	-	
3 – 7	107/6	4.7	0.56 – 38.9		2.4	0.28 – 20.8	
≥ 8	122/40	34.1	4.7 – 248.4		7.7	0.95 – 62.1	
Original variable	318/47	1.10	1.08 – 1.13	< 0.001	1.05	1.02 – 1.09	0.002
**Event Free Survival**				< 0.001			< 0.001
0 – 2	89/6	1 (ref.)	-		1 (ref.)	-	
3 – 7	107/15	2.1	0.81 – 5.4		2.1	0.75 – 5.9	
≥ 8	122/63	9.6	4.2 – 22.3		7.4	2.7 – 20.5	
Original variable	318/84	1.07	1.05 – 1.09	< 0.001	1.04	1.02 – 1.07	0.002

N/O: Number of patients/Outcome (Deaths or Events). HR: Hazard Ratio. Multivariable analysis: HRs are adjusted by age, and stage at diagnosis. ref. = referent group. Original variable = original values of BPI non categorized to assess the linear trend.

Supplementary Table S4. Overall and Event Free Survival of 100 patients diagnosed between 1999 and 2020 with amplified *MYCN* status in relation to the BPI levels evaluated by the Cox model.

		Univariable analysis			Multivariable analysis		
**BPI**	**N/O**	**HR**	**95%CI**	***p***	**HR**	**95%CI**	***p***
**Overall Survival**				0.087			0.122
0 – 9	27/11	1 (ref.)	-		1 (ref.)	-	
10 – 13	37/15	0.87	0.40 – 1.9		0.85	0.38 – 1.9	
≥ 14	36/22	1.7	0.84 – 3.6		1.7	0.80 – 3.6	
Original variable	100/48	1.03	0.99 – 1.07	0.168	1.02	0.98 – 1.06	0.313
**Event Free Survival**				0.202			0.264
0 – 9	27/13	1 (ref.)	-		1 (ref.)	-	
10 – 13	37/16	0.79	0.38 – 1.7		0.75	0.35 – 1.6	
≥ 14	36/22	1.5	0.74 – 2.9	0.329	1.4	0.70 – 2.9	
Original variable	100/51	1.02	0.98 – 1.05	0.329	1.01	0.97 – 1.05	0.558

N/O: Number of patients/Outcome (Deaths or Events). HR: Hazard Ratio. Multivariable analysis: HRs are adjusted by age, stage at diagnosis and treatment era. ref. = referent group. Original variable = original values of BPI non categorized to assess the linear trend.

Supplementary Table S5. Overall and Event Free Survival of 237 patients diagnosed between 1999 and 2020 with localized stage at diagnosis in relation to the BPI levels evaluated by the Cox model.

		Univariable analysis			Multivariable analysis		
**BPI**	**N/O**	**HR**	**95%CI**	***p***	**HR**	**95%CI**	***p***
**Overall Survival**				< 0.001			0.003
0 – 2	81/1	1 (ref.)	-		1 (ref.)	-	
3 – 5	76/1	1.07	0.07 – 17.2		1.1	0.07 – 18.6	
≥ 6	80/23	22.9	3.1 – 169.4		11.2	1.4 – 92.0	
Original variable	237/25	1.13	1.09 – 1.18	< 0.001	1.14	1.07 – 1.21	< 0.001
**Event Free Survival**				< 0.001			< 0.001
0 – 2	81/5	1 (ref.)	-		1 (ref.)	-	
3 – 5	76/7	1.5	0.47 – 4.7		1.6	0.49 – 5.0	
≥ 6	80/33	8.2	3.2 – 20.9		7.3	2.6 – 20.1	
Original variable	237/45	1.11	1.08 – 1.14	< 0.001	1.10	1.05 – 1.15	< 0.001

N/O: Number of patients/Outcome (Deaths or Events). HR: Hazard Ratio. Multivariable analysis: HRs are adjusted by *MYCN* status, age at diagnosis and treatment era. ref. = referent group. Original variable = original value of BPI non categorized to assess the linear trend.

Supplementary Table S6. Overall and Event Free Survival of 166 patients diagnosed between 1999 and 2020 with stage M at diagnosis in relation to the BPI levels evaluated by the Cox model.

		**Univariable analysis**			**Multivariable analysis**		
**BPI**	**N/O**	**HR**	**95%CI**	***p***	**HR**	**95%CI**	***p***
**Overall Survival**				n.e.			n.e.
0 – 9	42/13	1 (ref.)	-		1 (ref.)	-	
10 – 16	66/28	1.7	0.88 – 3.3		1.5	0.75 – 2.8	
≥ 17	58/31	2.2	1.1 – 4.2		2.3	1.1 – 4.5	
Original variable	166/72	1.02	1.00 – 1.05	0.077	1.02	1.00 – 1.05	0.090
**Event Free Survival**				n.e.			n.e.
0 – 9	42/18	1 (ref.)	-		1 (ref.)	-	
10 – 16	66/34	1.4	0.77 – 2.4		1.2	0.67 – 2.1	
≥ 17	58/38	1.6	0.91 – 2.8		1.5	0.81 – 2.7	
Original variable	166/90	1.01	0.99 – 1.04	0.257	1.00	0.98 – 1.03	0.464

N/O: Number of patients/Outcome (Deaths or Events). HR: Hazard Ratio. Multivariable analysis: HRs are adjusted by *MYCN* status, age at diagnosis and treatment era. n.e. = not evaluable due to the violation of the proportional hazards' assumption. ref. = referent group. Original variable = original value of BPI non categorized to assess the linear trend.

Supplementary Table S7. Overall and Event Free Survival of 256 patients diagnosed between 1999 and 2020 with segmental chromosomal aberrations at diagnosis in relation to the BPI levels evaluated by the Cox model.

		**Univariable analysis**			**Multivariable analysis**		
**BPI**	**N/O**	**HR**	**95%CI**	***p***	**HR**	**95%CI**	***p***
**Overall Survival**				< 0.001			0.003
0 – 9	68/14	1 (ref.)	-		1 (ref.)	-	
10 – 15	87/28	1.6	0.84 – 3.1		1.1	0.57 – 2.1	
≥ 16	101/49	3.0	1.6 – 5.4		2.3	1.2 – 4.3	
Original variable	256/91	1.04	1.02 – 1.07	< 0.001	1.03	1.00 – 1.06	0.025
**Event Free Survival**				< 0.001			0.002
0 – 9	68/22	1 (ref.)	-		1 (ref.)	-	
10 – 15	87/36	1.3	0.76 – 2.2		1.0	0.60 – 1.8	
≥ 16	101/60	2.2	1.4 – 3.6		1.8	1.0 – 3.0	
Original variable	256/118	1.03	1.01 – 1.05	0.003	1.02	1.00 – 1.04	0.107

N/O: Number of patients/Outcome (Deaths or Events). HR: Hazard Ratio. Multivariable analysis: HRs are adjusted by *MYCN* status, stage, age at diagnosis and treatment era. n.e. = not evaluable due to the violation of the proportional hazards' assumption. ref. = referent group. Original variable = original value of BPI non categorized to assess the linear trend.
